# Task Inhibition and Response Inhibition in Older vs. Younger Adults: A Diffusion Model Analysis

**DOI:** 10.3389/fpsyg.2016.01722

**Published:** 2016-11-15

**Authors:** Stefanie Schuch

**Affiliations:** Institute of Psychology, RWTH Aachen UniversityAachen, Germany

**Keywords:** task switching, inhibition, n−2 task repetition costs, response-repetition effects, aging, diffusion modeling

## Abstract

Differences in inhibitory ability between older (64–79 years, *N* = 24) and younger adults (18–26 years, *N* = 24) were investigated using a diffusion model analysis. Participants performed a task-switching paradigm that allows assessing n−2 task repetition costs, reflecting inhibitory control on the level of tasks, as well as n−1 response-repetition costs, reflecting inhibitory control on the level of responses. N−2 task repetition costs were of similar size in both age groups. Diffusion model analysis revealed that for both younger and older adults, drift rate parameters were smaller in the inhibition condition relative to the control condition, consistent with the idea that persisting task inhibition slows down response selection. Moreover, there was preliminary evidence for task inhibition effects in threshold separation and non-decision time in the older, but not the younger adults, suggesting that older adults might apply different strategies when dealing with persisting task inhibition. N−1 response-repetition costs in mean RT were larger in older than younger adults, but in mean error rates tended to be larger in younger than older adults. Diffusion-model analysis revealed longer non-decision times in response repetitions than response switches in both age groups, consistent with the idea that motor processes take longer in response repetitions than response switches due to persisting response inhibition of a previously executed response. The data also revealed age-related differences in overall performance: Older adults responded more slowly and more accurately than young adults, which was reflected by a higher threshold separation parameter in diffusion model analysis. Moreover, older adults showed larger non-decision times and higher variability in non-decision time than young adults, possibly reflecting slower and more variable motor processes. In contrast, overall drift rate did not differ between older and younger adults. Taken together, diffusion model analysis revealed differences in overall performance between the age groups, as well as preliminary evidence for age differences in dealing with task inhibition, but no evidence for an inhibitory deficit in older age.

## Introduction

According to the prominent “inhibition deficit hypothesis,” inhibitory functions deteriorate in older age (e.g., Hasher et al., 1999, 2007). To date, the evidence for an inhibition deficit in older age is mixed; it seems that different forms of inhibition need to be distinguished (e.g., Andrés et al., 2008; Germain and Colette, 2008; Borella et al., 2009; Anguera and Gazzaley, 2012).

Different paradigms have been developed in cognitive psychology to investigate inhibitory functions, many of which assess “low-level” inhibitory functions such as inhibition of previously attended stimulus locations (e.g., Taylor and Klein, 1998; Wang and Klein, 2009), inhibition of previously ignored stimuli (e.g., Fox, 1995; May et al., 1995; Tipper, 2001), or the stopping of ongoing responses (e.g., Verbruggen and Logan, 2008). The present study focuses on “higher-level” inhibitory functions that are involved in task switching performance, facilitating flexible switching between different tasks. Specifically, task inhibition and response inhibition in task switching are being investigated, assessing potential age-related differences in these inhibitory functions.

To investigate the ability to inhibit a previous task that is no longer relevant, a task-switching paradigm has been developed measuring “n−2 task repetition costs” (Mayr and Keele, 2000; for reviews, see Koch et al., 2010; Gade et al., 2014). The basic idea is that switching from task A to task B involves inhibition of the no longer relevant task A. When switching back to A after just one intermediate trial (ABA task sequence), task A is still inhibited and this persisting inhibition needs to be overcome, leading to performance costs, relative to task sequences where at least two intermediate trials have occurred before switching back to task A, and hence there is less persisting inhibition of A (CBA task sequence).

Another inhibitory function involved in task-switching performance is response inhibition, serving to prevent perseveration of a response that has already been executed (e.g., Rogers and Monsell, 1995; Houghton and Tipper, 1996; Druey and Hübner, 2008). Response inhibition can be measured by assessing response-repetition costs in task-switching paradigms (e.g., Hübner and Druey, 2006; Koch et al., 2011; Druey, 2014). Repeating the response from the previous trial takes longer than switching the response, due to persisting response inhibition. This response-repetition cost only becomes apparent in task-switch trials, when the same response needs to be repeated in a different task context. In task repetitions, the response-repetition cost is overcompensated by other cognitive processes, such as category priming or episodic binding (cf. Oberauer et al., 2013; Druey, 2014).

On the basis of the inhibition-deficit-theory of aging (see also Dempster, 1992; Hasher et al., 1999, 2007; Gazzaley, 2012), one would expect task inhibition and response inhibition to be diminished in older as compared to younger adults. So far, however, empirical support for such age-related diminution of task inhibition and response inhibition has not been reported. Mayr (2001) compared n−2 task repetition costs and response-repetition effects in young vs. older adults. If anything, older adults showed even larger n−2 task repetition costs than younger adults. With respect to response-repetition effects, Mayr (2001) found age differences in task repetitions, with larger response-repetition benefit in older than younger adults. Response-repetition costs in task switches were small and were not compared directly between the age groups, because response inhibition was not in the focus of interest in that study. Lawo et al. (2012) also looked at n−2 task repetition costs in older vs. younger adults, and found n−2 task repetition costs of similar size in both age groups (see also Li and Dupuis, 2008). In both Mayr's (2001) and Lawo et al.'s (2012) study, the inhibition effects were observed in mean RT data; inhibition effects in mean error rates were small and non-significant. Pettigrew and Martin (2015) observed increased n−2 task repetition costs in older as compared to younger adults when computing “rate residual scores,” which are a composite measure of RT and error rates that controls for potential age differences in processing speed (cf. Hughes et al., 2014). Response-repetition costs were not analyzed in this latter study. Hence, if anything, task inhibition has been found to be larger in older than younger adults, and response inhibition has not been systematically compared between older vs. younger adults.

In the above-mentioned studies, the data were analyzed by computing mean performance per experimental condition (e.g., mean RT in ABA vs. CBA trials), or by comparing the residuals of a regression of the more difficult ABA condition on the easier CBA condition (Pettigrew and Martin, 2015). It is possible that subtle differences in the shape of RT *distributions* of older vs. younger adults are not detected by such approaches. A more exhaustive analysis of choice-RT data can be obtained by applying the diffusion model (Ratcliff, 1978; Ratcliff and McKoon, 2008; Ratcliff et al., 2015, 2016), taking into account the response time distributions of both correct and error responses. The model parameters can be interpreted in terms of cognitive processes, making it possible to draw inferences about the cognitive mechanisms underlying age differences in behavioral performance (cf. Matzke and Wagenmakers, 2009; Voss et al., 2013, 2015).

**Figure 1:**
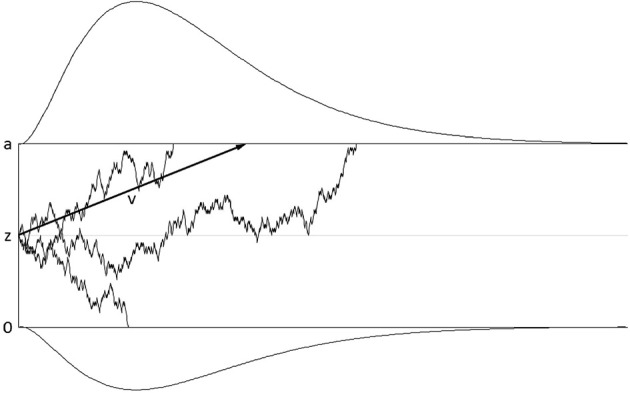
**Illustration of the basic diffusion model**. Time passes from left to right. The upper and lower thresholds represent the amount of evidence necessary to select one or the other response alternative, respectively. The upper threshold corresponds to the correct response alternative, the lower threshold to the wrong response alternative. Evidence accumulation starts at starting point *z* and continues until one of the thresholds is reached. Evidence accumulation is influenced by random noise; the average rate of evidence accumulation (drift rate ν) is shown by the black arrow. The drifts of three individual trials are shown. Reaction time is a linear function of the duration of the drift process; reaction time distributions for the correct and wrong response alternative are illustrated above and below the thresholds.

The diffusion model assumes that evidence for one or the other response alternative is accumulated until a threshold is reached, after which this response is executed (see Figure 1 for an illustration). In its simplest version, the model has three parameters: The speed of evidence accumulation is described by the drift rate parameter; the amount of evidence required before a response is selected is described by the threshold separation parameter; these two parameters determine the shape of the response time distribution. A third parameter subsumes all processes before and after the response selection process and is therefore called non-decision time parameter. Apart from these three basic parameters, the starting point can be varied as well, modeling biases toward one or the other response alternative. Moreover, variability in starting point, drift rate, and non-decision time can be introduced as additional parameters. Variability in starting point and drift rate have only small impact on the shape of the resulting response time distribution (cf. Voss et al., 2013); a recent study by Lerche and Voss (2016) showed that using a more parsimonious model with these variability parameters fixed to zero can be superior to more complex models. Variability in non-decision time has a larger impact on the shape of the distribution; therefore, it has been recommended to include non-decision time variability in the model in order to achieve stable parameter estimates (Voss et al., 2015; Lerche and Voss, 2016).

The diffusion model has been applied extensively to assess the effects of aging on performance in choice-RT tasks (e.g., Thapar et al., 2003; Ratcliff et al., 2006a,b, 2007, 2011; Spaniol et al., 2006; McKoon and Ratcliff, 2013; Ratcliff and McKoon, 2015). It is usually found that older adults respond more slowly, but also more accurately, than younger adults, which is reflected in a larger threshold separation parameter in older than younger adults in diffusion model analysis. Moreover, motor processes have been found to be prolonged in older age, leading to increased non-decision time parameters in older compared to younger adults. In contrast, the quality of information on which the decision is based is often as good in older as in younger adults, as reflected in comparable drift rates across age groups.

Regarding n−2 task repetition costs in young adults, a previous study from our lab has found the ABA–CBA difference to be reflected in the drift rate, with smaller drift rate in ABA than CBA trials (Schuch and Konrad, under review). This finding is in line with previous diffusion-model studies of task-switching performance, where carry-over effects of previous task sets have been found to be reflected in drift rate (Schmitz and Voss, 2012, 2014). Because n−2 task repetition costs are thought to be a measure of persisting inhibition of a previously abandoned task set, they, too, constitute a carry-over effect of previous task sets. Interestingly, Schuch and Konrad (under review) showed that n−2 task repetition costs in a group of 9–11 year old children were not reflected in drift rate, but in non-decision time, suggesting that different cognitive processes might be underlying n−2 task repetition costs in children vs. young adults. In light of these findings the question arises as to whether n−2 task repetition costs in older vs. younger adults might result from partly different cognitive processes as well, as could be revealed by diffusion model analysis. Regarding response-repetition costs in task switching, these have not been systematically investigated using diffusion model analysis. It is conceivable that response inhibition is reflected in non-decision time, slowing motor processes in response-repetition relative to response-switch trials.

In the present study, task inhibition and response inhibition were assessed in a group of older and younger adults. First, mean RTs and error rates were analyzed. Because RTs were expected to be considerably slower in older than younger adults, log-transformed RTs were analyzed in addition to raw RTs. By computing the inhibition effects on the basis of mean log RTs, age-related differences in overall cognitive speed can be accounted for (e.g., Kray and Lindenberger, 2000; Salthouse and Hedden, 2002; as a side effect, the log transformation also reduces skewness of the RT distribution, e.g., Ratcliff, 1993). Second, a diffusion model analysis was performed on the raw data in order to investigate which cognitive processes underlie the inhibition effects in the two age groups. Based on previous studies, it was predicted that task inhibition is reflected in the drift rate parameter, at least in young adults. Response inhibition was predicted to be reflected in the non-decision time parameter, reflecting prolonged motor processes. Comparing diffusion model parameters of young vs. old adults will allow investigating potential age differences in task inhibition and response inhibition.

## Methods

### Participants

Twenty-four older adults (range 64–79 years; mean age 71.7 years, *SD* 4.0; 12 female; 12 male) were recruited from the voluntary participants list of the Cognitive and Experimental Unit at Institute of Psychology, RWTH Aachen University, and received 8 Euros for participation. All older adults were retired; the period of retirement varied from 1 to 17 years (mean 9.3 years, *SD* 4.6). The DemTect (Kessler et al., 2000) was administered to control for potential signs of dementia; the participants' DemTect values varied between 15 and 18 (mean 17.4; *SD* 0.8), and hence were all within the normal range. (The maximum DemTect value is 18; values above 13 are considered normal in people of 60 years or older).

Twenty-four young adults (range 18–26 years; mean age 21.0 years, *SD* 2.6; 12 female; 12 male) were recruited from the Aachen area; they were either students, or friends of students, of RWTH Aachen University, and received 8 Euros or partial course credits for participation. One participant in the young adult group was replaced because of showing a two-peaked RT distribution. (The overall RT distribution, as well as the separate distributions of ABA and CBA, and of response repetitions and response switches, all showed two peaks in this participant, possibly indicating that this person applied two different strategies when performing the experiment. The RT distributions of all other participants and conditions were all one-peaked).

The study was in accordance with the Declaration of Helsinki. All participants gave written informed consent to participate in the study.

### Stimuli, tasks, and responses

The stimuli were standardized facial photographs of 20 young adults (20–30 years old) and 20 older adults (60–70 years old). Each portrait was presented inside a colored frame, with frame color indicating which task to perform. A blue frame indicated to categorize the person as male or female; a red frame to categorize the person as young or old; a yellow frame to categorize the emotional expression as happy or angry. The 40 faces consisted of 5 young male happy faces, 5 young male angry, 5 young female happy, 5 young female angry, 5 old male happy, 5 old male angry, 5 old female happy, 5 old female angry. The color frames (14.5 cm in height and 11 cm in width; frame line of 0.3 cm thickness) were presented centrally on a black computer screen. The portraits (14.1 cm in height size, 10.6 cm in width) were presented centrally inside the frames. The computer screen was situated about 50 cm in front of the participants. Participants responded by pressing one of two response keys on a German computer keyboard (the “x” and “,” keys, which are located just above the left and right end of the space bar, respectively) with their left or right index finger, respectively. Half of the participants in each age group responded to happy, young, and male, faces by pressing the left key, and to angry, old, and female faces by pressing the right key. To the other half of the participants, the reversed mapping was assigned (right for happy, young, male; left for angry, old, female). The paradigm was the same as in the study by Schuch and Konrad (under review; see Schuch et al., 2012, for further details of the stimulus material).

### Procedure

Participants were instructed orally by the experimenter; in addition, written instructions were presented on the screen. Participants were encouraged to respond as quickly and as accurately as possible. The experimenter stayed in the room over the whole period of the experiment. Participants completed four practice blocks of 60 trials each. In practice blocks 1–3, the tasks were practiced separately (gender categorization task in block 1, age categorization task in block 2, emotion categorization task in block 3). In practice block 4, all three tasks occurred in pseudo-random order.

The experimental phase consisted of four blocks of 60 trials each, which were separated by short breaks. Cues and stimuli occurred in pseudo-random order, with the following constraints. (1) Immediate task repetitions were not allowed. (2) Each task occurred equally often in each block. (3) There were roughly equal numbers of n−2 task repetitions and n−2 task switches per block. (4) Each of the 40 stimuli occurred six times during the experimental blocks, and six times during the practice phase. (5) Each stimulus was presented twice in the context of each task during the experimental blocks, and twice in the context of each task during the practice blocks. (6) The person presented in a particular trial n was never the same as the persons presented in trials n−1 and n−2. (7) There were roughly equal numbers of response repetitions and response switches from trial n−2 to n−1 within each block and within the ABA and CBA task sequences.

Every trial started with the presentation of a red, blue, or yellow frame for 500 ms, followed by the presentation of a photograph inside the frame. Frame and picture stayed on the screen until the left or right response key was pressed. Then the screen turned black for 1000 ms. If the wrong key was pressed, an error feedback occurred after 500 ms of blank screen and lasted for 1000 ms, after which the screen turned black again for another 500 ms.

### Design

For the analysis of task inhibition, a 2 × 2 design was applied with the independent variables Task Sequence (ABA vs. CBA) and Age Group (older vs. young adults). For the analysis of response inhibition, a 2 × 2 design was applied with the independent variables Response Transition (response repetition vs. response switch from trial n−1 to n) and Age Group (older vs. young adults). The two kinds of inhibition were analyzed separately in order to have a sufficient number of trials per condition for robust parameter estimation in the diffusion model analysis. For analysis of mean performance per experimental condition, the dependent variables were RTs, log RTs, and error rates. For diffusion model analysis, dependent variables were the parameters drift rate, threshold separation, non-decision time, and variability of non-decision time.

## Results

### Data filtering

The first two trials from each experimental block (which could not be classified as ABA or CBA) were removed from analysis, as well as the two trials following an error (to eliminate potential influences of error aftereffects). Outliers were removed as well; these were defined following the procedure recommended by Schmiedek and colleagues (Schmiedek et al., 2007; see also Steinhauser and Hübner, 2009; Moutsoupoulou and Waszak, 2012). That is, trials with RT faster than 200 ms were excluded, then trials with RT higher than four standard deviations above each participant's mean per experimental condition were defined as outliers. This process was repeated on the remaining trials until there were no further outliers. For analysis of mean RTs, error trials were excluded as well; for analysis of error rates and diffusion model analysis, error trials were included. For analysis of task inhibition, the mean number of trials per condition in young adults were 98.9 (*SD* 8.0; range 78–108) in ABA and 102.3 (*SD* 7.8; range 86–112) in CBA condition; in the older adults, there were 102.2 (*SD* 7.1; range 86–114) in ABA and 107.1 (*SD* 6.4; range 89–116) in CBA condition. For analysis of response inhibition, the mean number of trials per condition in young adults were 96.1 (*SD* 8.3; range 72–106) in response repetitions and 105.1 (*SD* 8.0; range 91–117) in response switches; in the older adults, there were 101.4 (*SD* 6.2; range 87–110) in response repetitions and 107.8 (*SD* 7.2; range 93–120) in response switches.

The analyses were performed on 24 young and 24 older adults. Because variability in the inhibition effects was large in diffusion model parameters, secondary analyses were conducted where participants with outlying inhibition effects in one or more of the model parameters were excluded (see Supplementary Figure 1). For the secondary analysis of task inhibition, this affected two young and six older adults; for response inhibition, this affected two young and two older adults. To foreshadow the results, the overall data pattern was similar in both types of analyses. Statistically, the pattern of main effects was the same in both types of analyses, but the interactions of inhibition effects and age group were only significant on the 5% level in the analysis where participants with outlying inhibition effects were excluded. The interpretation of the data pattern is solely based on this secondary analysis.

### Diffusion model analysis

#### Parameter settings

The software “fast-dm” (Voss and Voss, 2007; Voss et al., 2015) was used to estimate the four parameters drift rate (ν), threshold separation (*a*), non-decision time (*t*_0_), and variability of non-decision time (*s*_*t*0_). The starting point bias was set to 0.5 *a* (i.e., in the middle between the two thresholds); this was done because the thresholds were associated with correct and erroneous responses (cf. Schmitz and Voss, 2012, 2014). All other parameters implemented in fast-dm were set to zero in order to keep the model as parsimonious as possible; this has been shown to improve estimation of the main parameters (Lerche and Voss, 2016; van Ravenzwaaij et al., 2016). The four parameters ν, *a, t*_0_, and *s*_*t*0_ were estimated separately for each individual and each condition (ABA vs. CBA in the task-inhibition analysis; response repetition vs. response switch in the response-inhibition analysis).

#### Model fit

The Kolmogorov–Smirnow (KS) statistic provided by the fast-dm software did not reveal any significant deviations between empirical and estimated RT distributions, *p*s > 0.21 for the analysis of task inhibition, *p*s > 0.30 for the analysis of response inhibition, suggesting that the model fitted the data reasonably well for all participants and all conditions. For visual inspection of model fit, the cumulative density functions (cdfs) were computed for each individual and each condition, and plotted together with the *p*-values of the KS statistic (see Supplementary Figures 2, 3)^1^.

### Analysis of task inhibition

**Figure 2:**
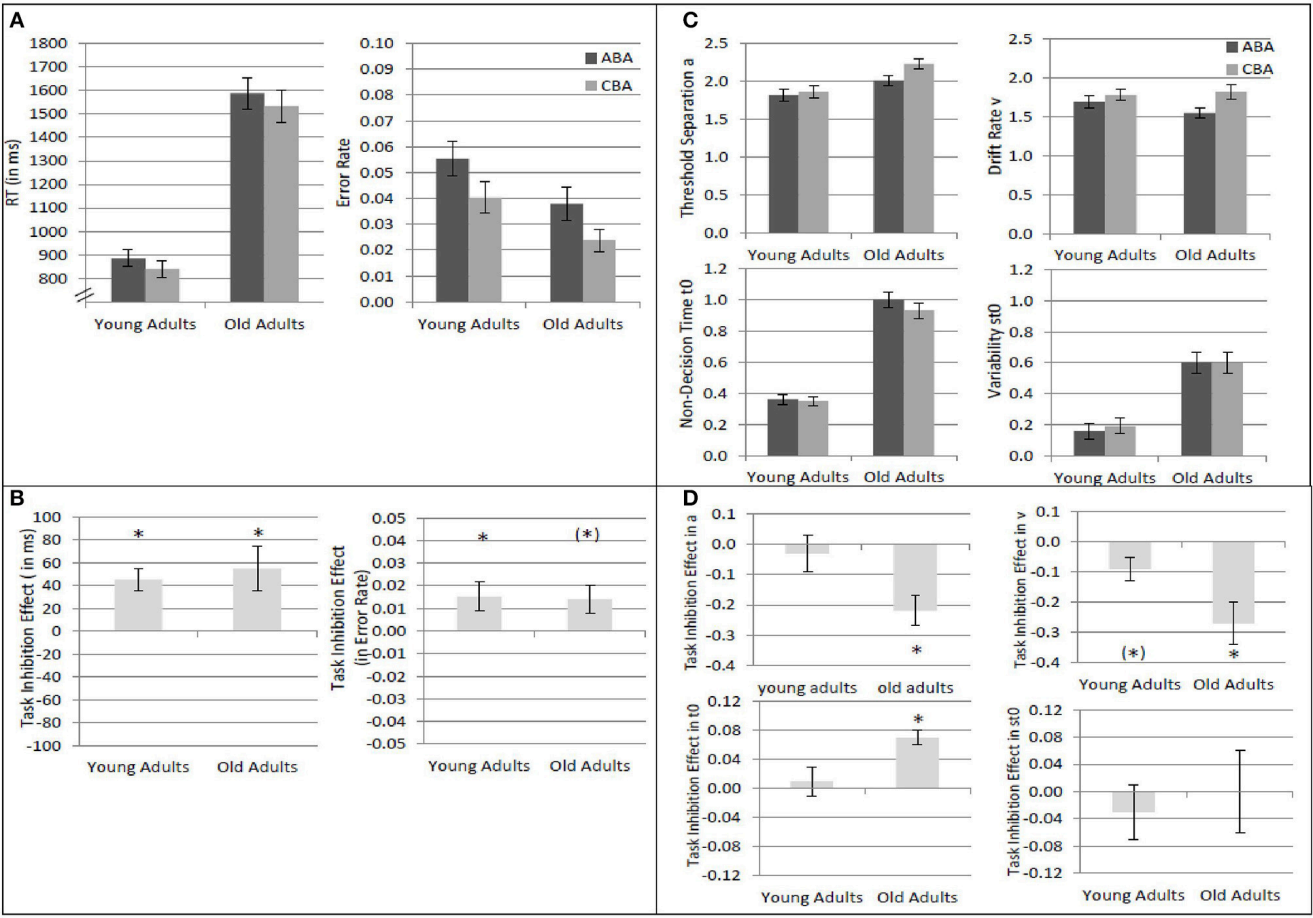
**Analysis of task inhibition (ABA vs. CBA task sequences) in young adults (18–26 years; ***N*** = 22) and older adults (64–79 years; ***N*** = 18). (A)** Mean reaction times and mean error rates in ABA and CBA trials. **(B)** Mean task inhibition effect (ABA–CBA) in reaction times and error rates. **(C)** Diffusion model parameters threshold separation *a*, drift rate ν, non-decision time *t*_0_, and variability of non-decision time *s*_*t*0_, separately for ABA and CBA trials, and young and older adults. Units on the y-axis represent the untransformed values as obtained by the fast-dm software (Voss and Voss, 2007; diffusion coefficient = 1.0). The units represent amount of evidence for *a*; evidence per time for ν; time (in s) for *t*_0_ and *s*_*t*0_. **(D)** Mean task inhibition effect (ABA–CBA) in diffusion model parameters. Error bars indicate 1 standard error of mean. ^*^ indicates significant task inhibition effect, i.e., *p* < 0.05 for the two-tailed *t*-test comparing ABA and CBA within each age group; (^*^) indicates *p* < 0.10 for the two-tailed *t*-test.

**Figure 3:**
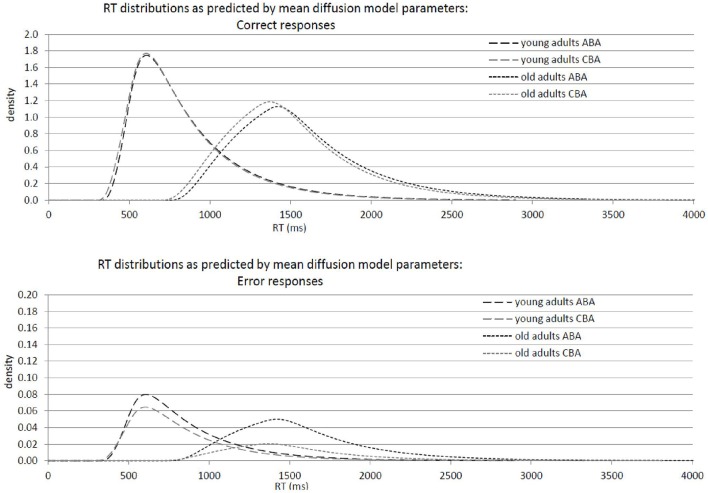
**Graphical illustration of response-time distributions resulting from mean diffusion model parameters in ABA and CBA conditions in young adults (18–26 years; ***N*** = 22) and older adults (64–79 years; ***N*** = 18)**. **Upper panel**: Distribution of correct responses. **Lower panel**: Distribution of error responses.

Results of the 2 × 2 ANOVAs with the independent variables Task Sequence (ABA vs. CBA) and Age Group (old vs. young adults) are described in Table 1. Specifically, Table 1A shows the ANOVAs including all participants; Table 1B shows the ANOVAs including only the participants with non-outlying task inhibition effects in model parameters. Figure 2 shows mean performance for ABA and CBA trials, as well as results from diffusion model analysis (all based on the analyses with non-outlying participants only). Figure 3 illustrates the RT distributions resulting from mean diffusion model parameters in ABA and CBA conditions in the two age groups. For illustrative purposes, the scale of error RT distributions is ten times larger than the scale of correct RT distributions.

**Table 1:** **Analysis of task inhibition: Results of the 2 × 2 ANOVAs with within-subjects variable Task Sequence (ABA, CBA) and between-subjects variable Age Group (young adults, older adults)**.

**(A) Analysis including all participants (24 young adults, 24 older adults)**.									
**Dependent measure**	**Main effect Age Group**			**Main effect Task Sequence**			**Interaction Task Sequence × Age Group**		
	***F*_(1, 46)_**	***p***	$\eta_{p}^{2}$	***F*_(1, 46)_**	***p***	$\eta_{p}^{2}$	***F*_(1, 46)_**	***p***	$\eta_{p}^{2}$
**MEAN PERFORMANCE**									
RT	56.72	<0.05	0.55	22.22	<0.05	0.33	<1.0	n.s.	
Log RT	94.75	<0.05	0.67	29.21	<0.05	0.39	<1.2	n.s.	
Error Rates	4.14	<0.05	0.08	8.42	<0.05	0.16	<1.0	n.s.	
**DIFFUSION MODEL PARAMETERS**									
*a*	7.38	<0.05	0.14	6.05	<0.05	0.12	2.42	= 0.13	0.05
ν	<1.0	n.s.		9.11	<0.05	0.17	2.68	= 0.11	0.06
*t*_0_	116.38	<0.05	0.72	4.61	<0.05	0.09	<1.0	n.s.	
*s*_*t*0_	25.15	<0.05	0.35	<1.0	n.s.		<1.0	n.s.	
**(B) Analysis including only participants with non-outlying task inhibition effects in model parameters (22 young adults, 18 older adults)**.									
**Dependent measure**	**Main effect Age Group**			**Main effect Task Sequence**			**Interaction Task Sequence × Age Group**		
	***F*_(1, 38)_**	***p***	$\eta_{p}^{2}$	***F*_(1, 38)_**	***p***	$\eta_{p}^{2}$	***F*_(1, 38)_**	***p***	$\eta_{p}^{2}$
**MEAN PERFORMANCE**									
RT	75.49	<0.05	0.67	18.10	<0.05	0.32	<1.0	n.s.	
Log RT	102.33	<0.05	0.73	21.75	<0.05	0.36	<1.0	n.s.	
Error Rates	4.59	<0.05	0.11	8.76	<0.05	0.19	<1.0	n.s.	
**DIFFUSION MODEL PARAMETERS**									
*a*	6.45	<0.05	0.15	8.87	<0.05	0.19	5.11	<0.05	0.12
ν	<1.0	n.s.		16.21	<0.05	0.30	3.78	= 0.06	0.09
*t*_0_	92.48	<0.05	0.71	11.14	<0.05	0.23	5.93	<0.05	0.14
*s*_*t*0_	23.46	<0.05	0.38	<1.0	n.s.		<1.0	n.s.	

Overall, mean RT was larger, and error rate was smaller, in older than younger adults. In diffusion model analysis, this was reflected by larger non-decision time, variability of non-decision time, as well as larger threshold separation in the older as compared to the younger adults. In contrast, drift rate did not differ between the age groups.

Regarding task inhibition, there were n−2 task repetition costs across both age groups in mean RT, mean log RT, and mean error rates, which did not differ statistically between older and younger adults. Diffusion model analysis revealed that the task inhibition effect was reflected in drift rate, threshold separation, and non-decision time, across both age groups. In ABA trials, drift rate was smaller, threshold separation smaller, and non-decision time was larger, than in CBA trials. This data pattern tended to be more pronounced in the old than young adults; the interactions of task inhibition and age group were not significant on a 5% alpha level when all participants were included, but were significant (or marginally significant) when only participants with non-outlying inhibition effects were included. These interactions were analyzed further by analyzing the age groups separately with *post-hoc* two-tailed *t*-tests. In the older adults, the task inhibition effect was significant in drift rate, *t*_(17)_ = 3.26, *p* < 0.01, threshold separation, *t*_(17)_ = 3.94, *p* < 0.01, and non-decision time, *t*_(17)_ = 4.09, *p* < 0.01. In the young adults, the task inhibition effect was marginally significant in drift rate, *t*_(21)_ = 2.05, *p* = 0.05, and in none of the other parameters, *t*s < 1.

### Analysis of response inhibition

**Figure 4:**
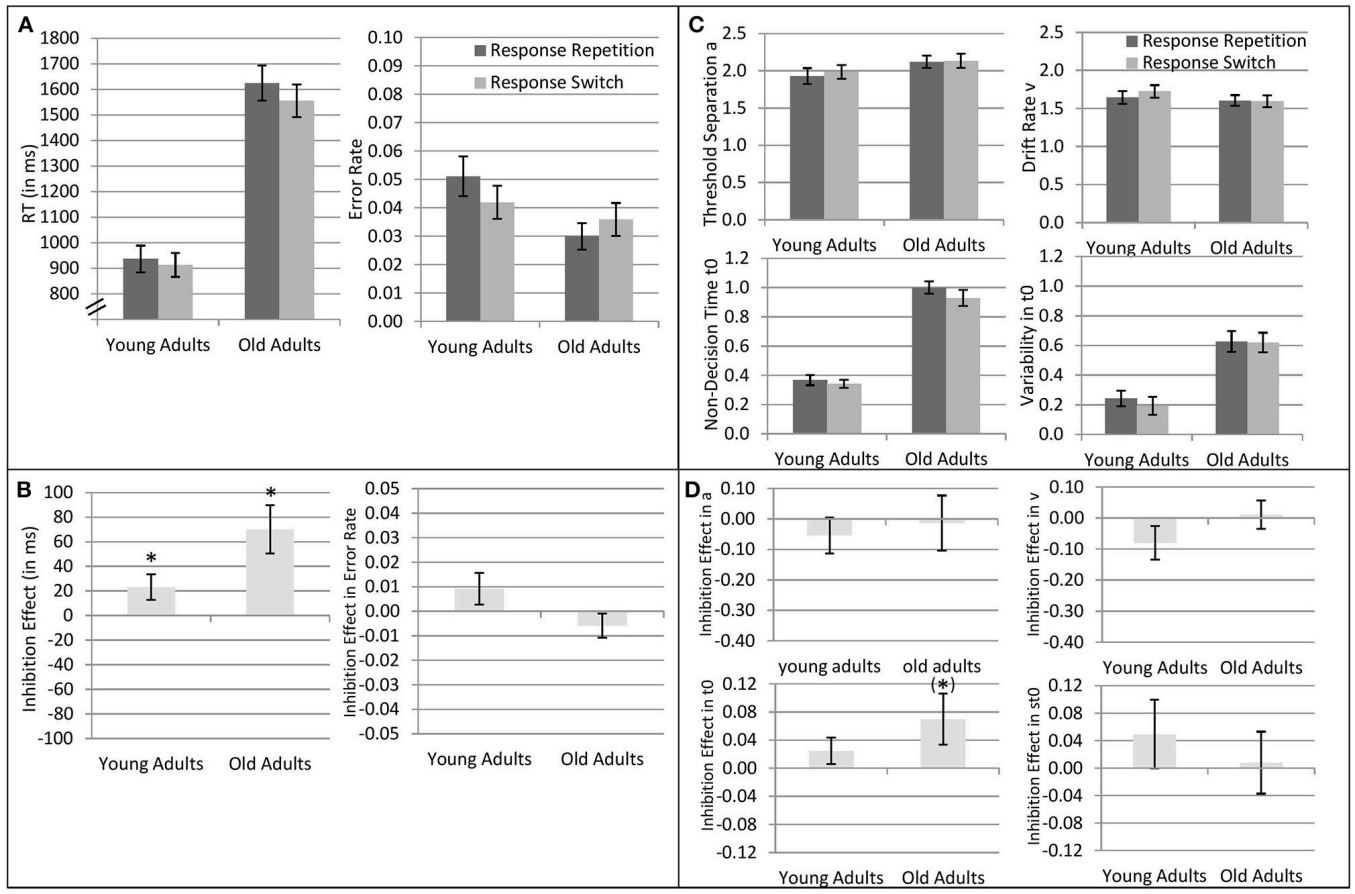
**Analysis of response inhibition (response repetitions vs. response switches from trial n−1 to ***n***) in young adults (18–26 years; ***N*** = 22) and older adults (64–79 years; ***N*** = 22)**. **(A)** Mean reaction times and mean error rates in response repetitions and switches. **(B)** Mean response inhibition effect (repetition-switch) in reaction times and error rates. **(C)** Diffusion model parameters threshold separation *a*, drift rate ν, non-decision time *t*_0_, and variability of non-decision time *s*_*t*0_, separately for response repetitions and switches, and young and older adults. Units on the y-axis represent the untransformed values as obtained by the fast-dm software (Voss and Voss, 2007; diffusion coefficient = 1.0). The units represent amount of evidence for *a*; evidence per time for ν; time (in s) for *t*_0_ and *s*_*t*0_. **(D)** Mean response inhibition effect (repetition-switch) in diffusion model parameters. Error bars indicate 1 standard error of mean. ^*^ indicates significant response inhibition effect, i.e., *p* < 0.05 for the two-tailed *t*-test comparing response repetition and response switch within each age group; (^*^) indicates *p* < 0.10 for the two-tailed *t*-test.

**Figure 5:**
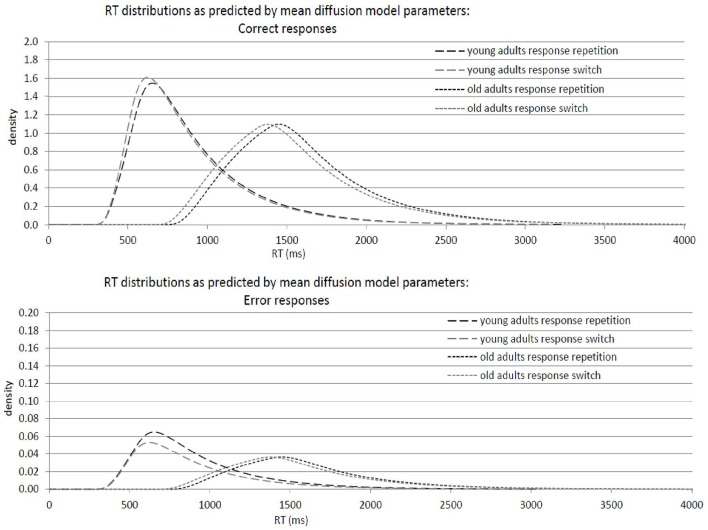
**Graphical illustration of response-time distributions resulting from mean diffusion model parameters in response repetitions and response switches in young adults (18–26 years; ***N*** = 22) and older adults (64–79 years; ***N*** = 22)**. **Upper panel:** Distribution of correct responses. **Lower panel:** Distribution of error responses.

Results of the 2 × 2 ANOVAs with the independent variables Response Transition (Response Repetition vs. Response Switch from n−1 to n) and Age Group (old vs. young adults) are described in Table 2. Table 2A shows the ANOVAs including all participants; Table 2B shows the ANOVAs including only the participants with non-outlying response inhibition effects in model parameters. Figure 4 shows mean performance in response repetitions and switches, as well as results from diffusion model analysis (all based on the analyses with non-outlying participants only). Figure 5 illustrates the RT distributions resulting from mean diffusion model parameters per condition and age group. For illustrative purposes, the scale of error RT distributions is ten times larger than the scale of correct RT distributions.

**Table 2:** **Analysis of Response Inhibition: Results of the 2 × 2 ANOVAs with within-subjects variable Response Transition (Response Repetition, Response Switch) and between-subjects variable Age Group (young adults, older adults)**.

**(A) Analysis including all participants (24 young adults, 24 older adults)**.									
**Dependent measure**	**Main effect Age Group**			**Main effect Response Transition**			**Interaction Response Transition × Age Group**		
	***F*_(1, 46)_**	***p***	$\eta_{p}^{2}$	***F*_(1, 46)_**	***p***	$\eta_{p}^{2}$	***F*_(1, 46)_**	***p***	$\eta_{p}^{2}$
**MEAN PERFORMANCE**									
RT	56.76	<0.05	0.55	12.83	<0.05	0.22	2.93	= 0.09	0.06
Log RT	94.69	<0.05	0.67	20.32	<0.05	0.31	1.84	= 0.18	0.04
Error Rates	4.40	<0.05	0.09	<1.0	n.s.		4.54	<0.05	0.09
**DIFFUSION MODEL PARAMETERS**									
*a*	4.79	<0.05	0.09	<1.6	n.s.		<1.0	n.s.	
ν	<1.7	n.s.		<1.0	n.s.		<1.0	n.s.	
*t*_0_	133.07	<0.05	0.74	7.91	<0.05	0.15	<1.6	n.s.	
*s*_*t*0_	26.20	<0.05	0.36	<1.2	n.s.		<1.0	n.s.	
**(B) Analysis including only participants with non-outlying response inhibition effects in model parameters (22 young adults, 22 older adults)**.									
**Dependent measure**	**Main effect Age Group**			**Main effect Response Transition**			**Interaction Response Transition × Age Group**		
	***F*_(1, 42)_**	***p***	$\eta_{p}^{2}$	***F*_(1, 42)_**	***p***	$\eta_{p}^{2}$	***F*_(1, 42)_**	***p***	$\eta_{p}^{2}$
**MEAN PERFORMANCE**									
RT	65.07	<0.05	0.61	17.68	<0.05	0.30	4.48	<0.05	0.10
Log RT	86.14	<0.05	0.67	19.57	<0.05	0.32	1.81	= 0.19	0.04
Error Rates	3.49	= 0.07	0.08	<1.0	n.s.		3.47	= 0.07	0.08
**DIFFUSION MODEL PARAMETERS**									
*a*	1.82	= 0.19	0.04	<1.0	n.s.		<1.0	n.s.	
ν	<1.0	n.s.		<1.0	n.s.		<1.6	n.s.	
*t*_0_	115.82	<0.05	0.73	4.88	<0.05	0.10	<1.2	n.s.	
*s*_*t*0_	21.97	<0.05	0.34	<1.0	n.s.		<1.0	n.s.	

The differences in overall performance obtained in the analysis of task inhibition were confirmed: Mean RT was larger, error rate smaller, the diffusion model parameters non-decision time, and variability of non-decision time were larger in older than younger adults; drift rate did not differ between the age groups. (Threshold separation was larger in older adults in the analysis including all participants, but this effect was not significant when the participants with outlying response inhibition effects were excluded).

There were n−1 response repetition costs across both age groups in mean RT and mean log RT, but not in error rates. Response-repetition costs in mean RT tended to be larger in older than younger adults, but in mean error rates, tended to be smaller in older than younger adults. Diffusion model analysis revealed that response-repetition costs were reflected in non-decision time across both age groups, with longer non-decision time in response repetitions than switches. (As can be seen from Figure 4, when the age groups were assessed separately, this effect was only marginally significant in the older adults, and not in the young adults.) The interaction of response inhibition and age group was not significant in any of the parameters.

### Combined analysis of task inhibition and response inhibition

In order to check for potential interactions between task inhibition and response inhibition, the data were also analyzed in a 2 × 2 × 2 ANOVA with the independent variables Task Sequence and Response Transition, as well as the between-subjects variable Age Group. The results are presented in Table 3; there were no significant interactions, neither of task inhibition and response inhibition, nor of task inhibition, response inhibition, and age group.

**Table 3:** **Analysis of Task Inhibition and Response Inhibition: Results of the 2 × 2 × 2 ANOVAs with within-subjects variables Task Sequence (ABA, CBA) and Response Transition (Response Repetition, Response Switch) and between-subjects variable Age Group (young adults, older adults)**.

**Dependent measure**	**Interaction Task Sequence × Response Transition**			**Interaction Task Sequence × Response Transition Age Group**		
	***F*_(1, 46)_**	***p***	**$\eta_{p}^{2}$**	***F*_(1, 46)_**	***p***	**$\eta_{p}^{2}$**
**MEAN PERFORMANCE**						
RT	<1.0	n.s.		<1.0	n.s.	
Log RT	<1.0	n.s.		<1.0	n.s.	
Error Rates	2.33	= 0.13	0.05	<1.0	n.s.	
**DIFFUSION MODEL PARAMETERS**						
*a*	2.22	= 0.14	0.05	<1.0	n.s.	
ν	<1.0	n.s.		<1.0	n.s.	
*t*_0_	<1.2	n.s.		<1.0	n.s.	
*s*_*t*0_	<1.0	n.s.		<1.0	n.s.	

*Only the interactions of interest are shown (two-way interaction of Task inhibition × Response Inhibition; three-way interaction of Task inhibition × Response inhibition × Age group). Analysis including all participants (24 young adults, 24 older adults)*.

## Discussion

The present study set out to investigate potential differences in inhibitory ability between younger and older adults. Two kinds of higher-level inhibition were investigated: task inhibition and response inhibition. Both effects were measured in a task-switching paradigm, where participants switched between three different face categorization tasks and every trial constituted a task switch. Task inhibition was measured as the difference between task sequences of type ABA (n−2 task repetition) vs. CBA (n−2 task switch); response inhibition was measured as the difference between response repetitions vs. response switches from trials n−1 to n. In addition to analysis of mean performance, diffusion modeling was applied, providing a more fine-grained picture of potential age differences in task inhibition and response inhibition. The results showed differences in overall performance between the age groups, but no evidence for reduced inhibitory ability in older adults, neither in mean performance nor in diffusion model parameters. These findings are discussed in more detail below.

### Overall performance

Regarding overall performance, older adults showed larger mean RTs, and smaller error rates, than younger adults, a finding that has long been known in the literature on aging (e.g., Rabitt, 1979; Salthouse, 1979; Smith and Brewer, 1995). In diffusion model analysis, this was reflected by a trend for larger threshold separation in older than younger adults (significant in the task-inhibition analysis, but not in the response-inhibition analysis). The threshold separation parameter can be interpreted as a marker of speed-accuracy trade off, and previous research has shown repeatedly that older adults emphasize accuracy over speed more than do younger adults (Ratcliff et al., 2007, 2010, 2011; Starns and Ratcliff, 2010, 2012; Ratcliff and McKoon, 2015). Moreover, non-decision time and variability of non-decision time were larger in older than younger adults. The larger non-decision time could indicate that stimulus encoding (Madden et al., 2009) and/or motor processes (Voss et al., 2004; Ratcliff et al., 2006a) are slower in older than younger adults; it could also be that task preparation takes longer in older than younger adults (Karayanidis et al., 2009; Schmitz and Voss, 2012, 2014). Other than threshold separation and non-decision time, drift rate did not differ between the age groups; that is, the quality of the accumulated evidence was of similar size in older and younger adults. This is in line with other aging studies, where drift rate has been found to be similar for younger and older adults across a wide range of tasks, such as signal detection tasks (Ratcliff et al., 2001), lexical decision tasks (e.g., Ratcliff et al., 2004), or item recognition memory tasks (e.g., Ratcliff et al., 2010, 2011; Ratcliff and McKoon, 2015). Interestingly, older adults differ from children in this respect, with children showing smaller drift rates than young adults in lexical decision (Ratcliff et al., 2012) and task-switching (Schuch and Konrad, under review) paradigms. This suggests that evidence accumulation is noisier in children than young adults, but is of similar quality in young and older adults.

### Task inhibition

Regarding task inhibition, n−2 task repetition costs were obtained across both age groups in mean RT, mean log RT, and mean error rates, which did not differ statistically between older and younger adults, confirming previous findings (Mayr, 2001; Lawo et al., 2012). Diffusion model analysis revealed that the task inhibition effect was reflected in drift rate, in line with another study from our lab (Schuch and Konrad, under review). Specifically, task inhibition was reflected in smaller drift rate in trials with more persisting inhibition (ABA) than in trials with less persisting inhibition (CBA), a finding fitting well with previous research suggesting that the task inhibition effect is mainly due to prolonged response selection in ABA relative to CBA trials (Schuch and Koch, 2003; Koch et al., 2010). This finding is also in line with diffusion-model studies of task-switching performance suggesting that carry-over effects from previous tasks affect drift rate (Schmitz and Voss, 2012, 2014). The inhibition effect in drift rate occurred in both age groups, and tended to be more pronounced in older than young adults. That is, the data clearly do *not* show a reduced inhibition effect in drift rate in older adults, as has been observed in children (Schuch and Konrad, under review), suggesting that inhibition of task-specific stimulus-response associations is at least as strong in older adults as in young adults.

Moreover, in the older but not the young adults, the task inhibition effect was also reflected in threshold separation and non-decision time, with smaller threshold separation and larger non-decision time in ABA than CBA trials. This could possibly mean that older adults engage in more advance task preparation in ABA than CBA, task preparation continues after stimulus onset, leading to longer non-decision time in ABA than CBA. This increased task preparation in ABA than CBA might involve a lowering of the response thresholds, as is reflected in smaller threshold separation in ABA than CBA. That is, older adults might apply different strategies than younger adults when performing the task-switching paradigm.

Although still speculative at this point, it could thus be the case that the comparable task inhibition effect obtained by analysis of mean performance is based on different strategies in young and older adults. The particular strategy applied might depend on the experimental setting; for instance, if emphasized in the instructions that advance task preparation is essential for performing the experiment, older adults might follow these instructions more closely than younger adults, and might hence engage in more task preparation. Differences in strategy could also be a possible reason for diverging findings in the literature (cf. Koch et al., 2010).

### Response inhibition

Regarding response inhibition, n−1 response repetition costs were obtained across both age groups in mean RT and mean log RT, but not in error rates. Response-repetition costs in mean RT tended to be larger in older than younger adults, but in mean error rates, they were smaller in older than younger adults. Diffusion model analysis revealed that response-repetition costs were reflected in non-decision time across both age groups, with longer non-decision time in response repetitions than switches. This is in line with the idea that in both age groups, persisting response inhibition slows down motor processes when this response needs to be executed again. (Although less likely, it is also possible that response inhibition slows down task preparation or stimulus encoding processes, given that the non-decision time parameter subsumes a whole range of cognitive processes, cf. Schmitz and Voss, 2012). No significant age differences in response-repetition costs were obtained in any of the parameters.

### Conclusion

Analysis of mean RTs and error rates revealed reliable task inhibition and response inhibition effects, but no consistent age-related differences in these inhibition effects, confirming previous studies. Diffusion model analysis revealed that persisting task inhibition slowed response selection, whereas persisting response inhibition slowed motor processes, in both older and younger adults. There was some preliminary evidence for strategic differences between young and older adults in dealing with persisting task inhibition; the older but not the young adults seemed to engage in more task preparation, and lower the response thresholds, in trials with persisting inhibition. No age-related differences in response inhibition were obtained in any of the parameters. In sum, diffusion model analysis did not reveal any evidence for an inhibitory deficit in older adults; rather, inhibitory ability on the task and response level in older adults was at least as strong as in younger adults; if anything, older adults might apply different strategies for overcoming persisting inhibition.

## Author contributions

SS planned and designed the study, programmed the experiment, analyzed and interpreted the data, and wrote the manuscript.

## Funding

Part of this research/SS was supported by a grant within the Priority Program, SPP 1772 from the German Research Foundation (Deutsche Forschungsgemeinschaft, DFG), grant no SCHU 3046/1-1.

### Conflict of interest statement

The author declares that the research was conducted in the absence of any commercial or financial relationships that could be construed as a potential conflict of interest.

## Footnotes

^1^Note that the standard criterion of *p* < 0.05 for the KS statistic to indicate poor model fit might not be ideally suited for all experimental settings. When trial numbers are relatively small (such as in the present study, where there are about 100 observations per condition), the power to detect misfits is relatively small. In contrast, when trial numbers are very large, even small misfits will reveal a significant *p* value of the KS statistic. One way to overcome this problem would be to run simulations in order to define an appropriate criterion adapted to the specific experimental setting (Voss et al., 2013). In the present study, we checked whether excluding all participants with *p* values smaller than.40 in the KS statistic would change the data pattern; it did not. Therefore, it was assumed that the model fitted the data sufficiently well for all participants.
