# Supplementary figures and images for: Task Inhibition and Response Inhibition in Older vs. Younger Adults: A Diffusion Model Analysis
Source: Front Psychol. 2016 Nov 15;7:1722. doi: 10.3389/fpsyg.2016.01722 (PMC5108792; doi:10.3389/fpsyg.2016.01722)

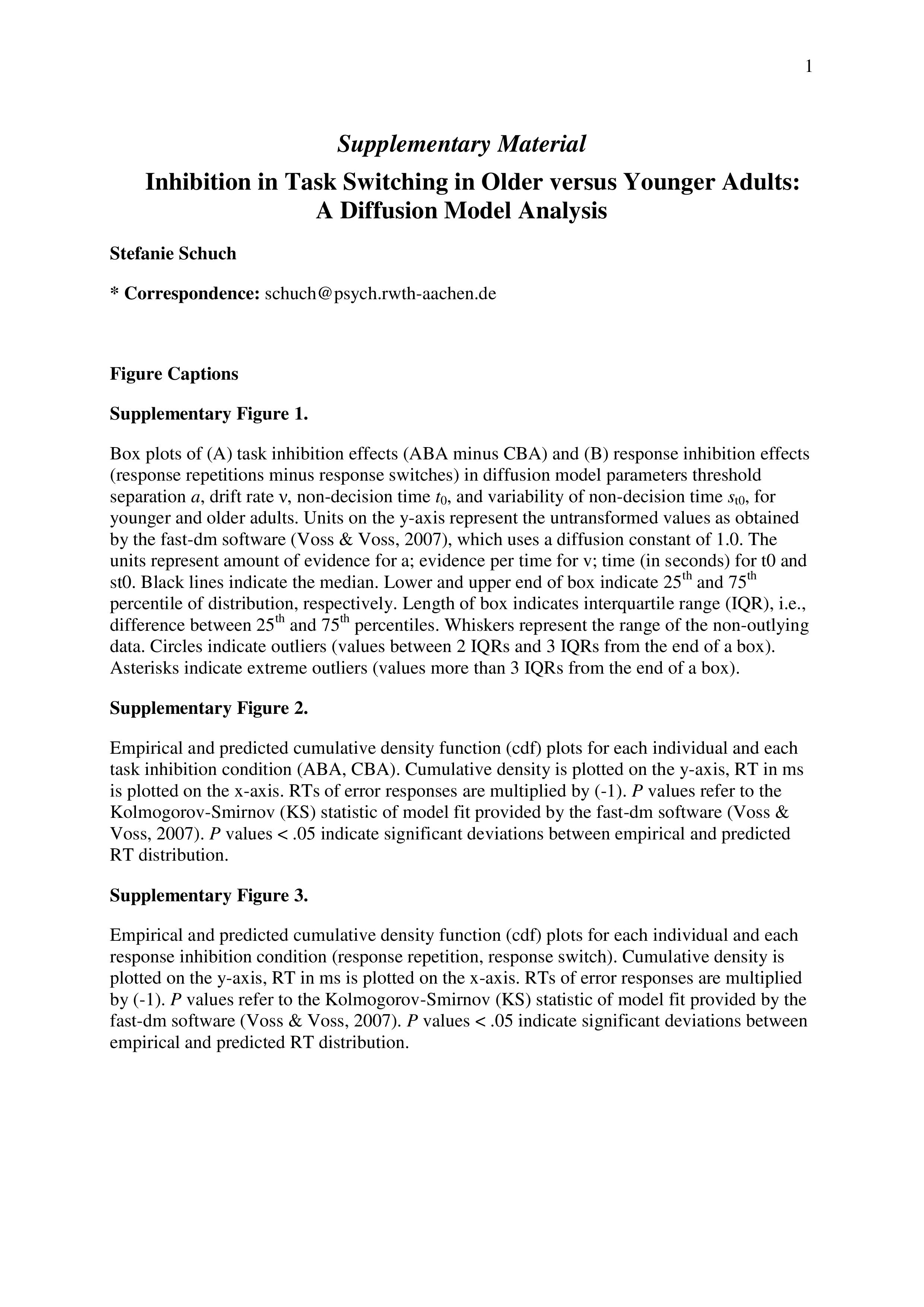

Supplement: Supplementary file 1 [file Image1.JPEG]

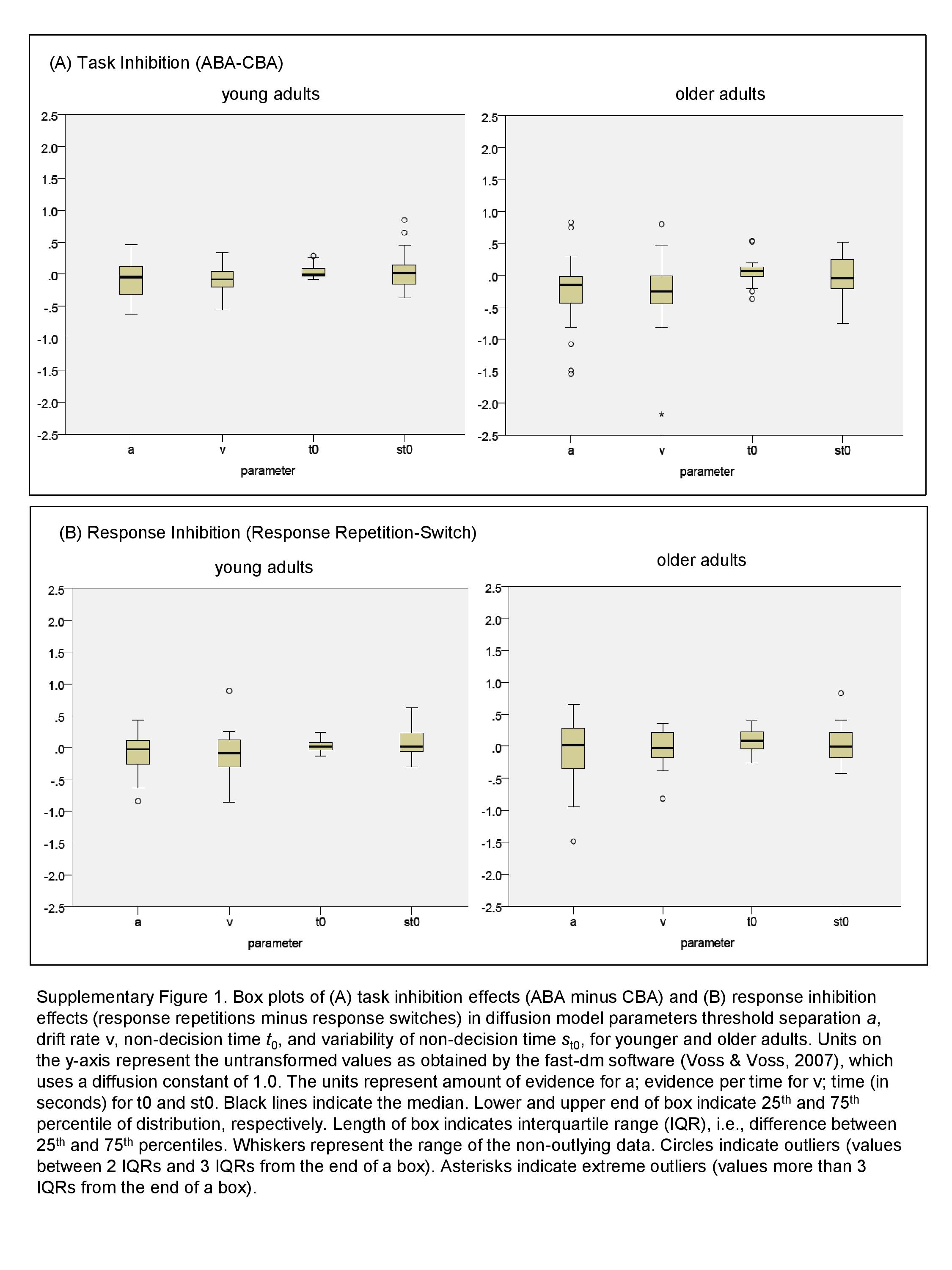

Supplement: Supplementary file 2 [file Image2.JPEG]

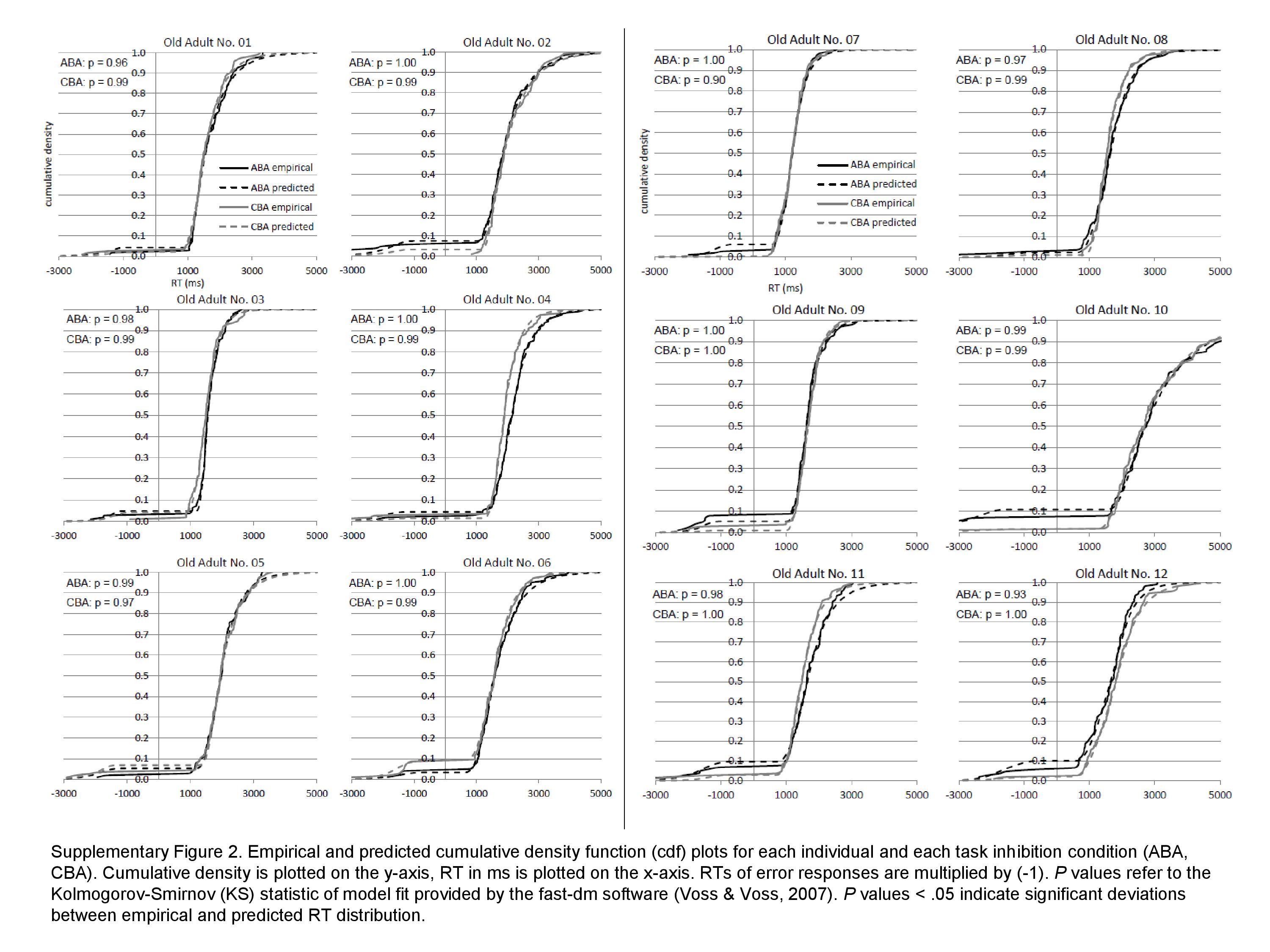

Supplement: Supplementary file 3 [file Image3.JPEG]

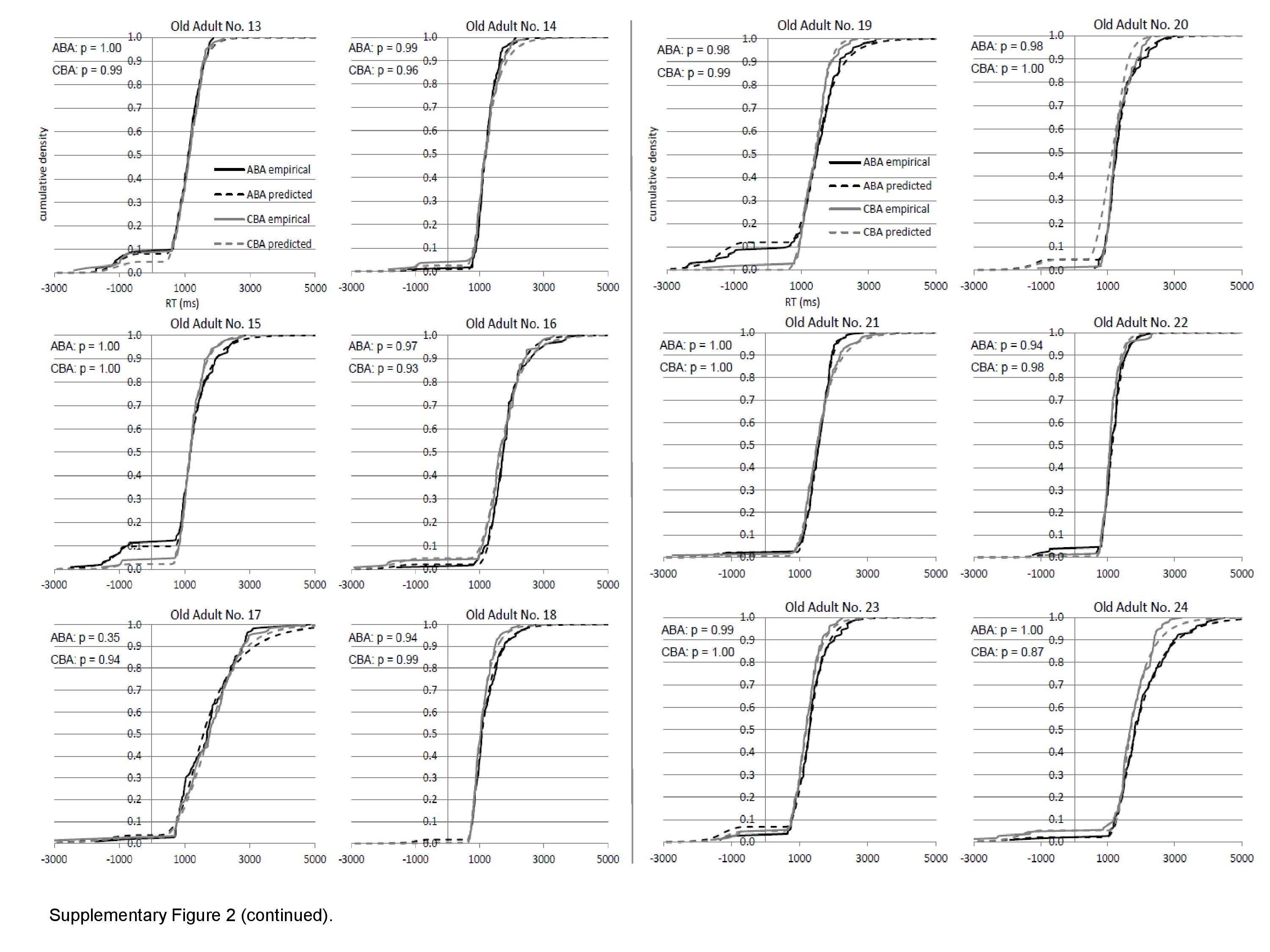

Supplement: Supplementary file 4 [file Image4.JPEG]

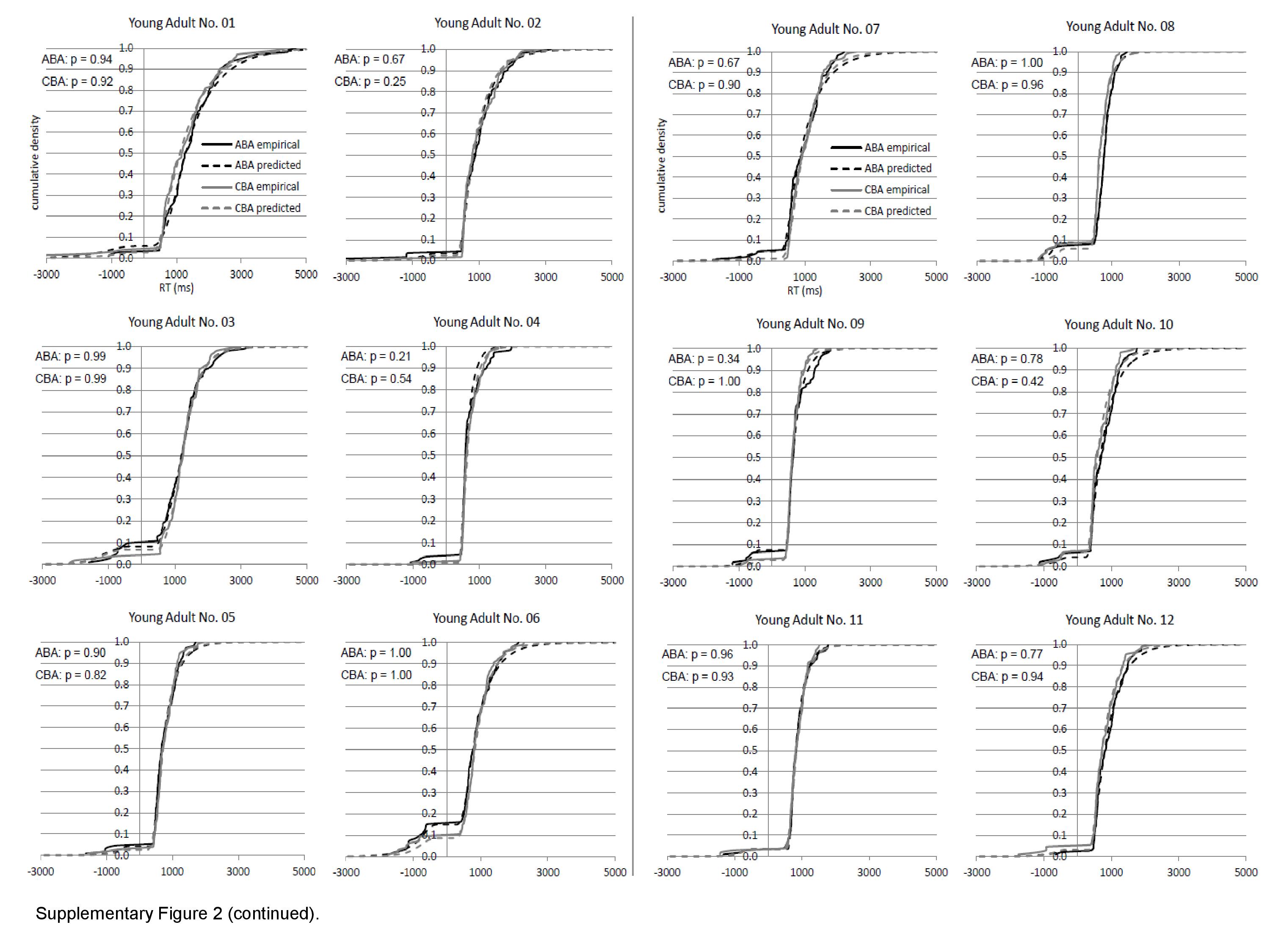

Supplement: Supplementary file 5 [file Image5.JPEG]

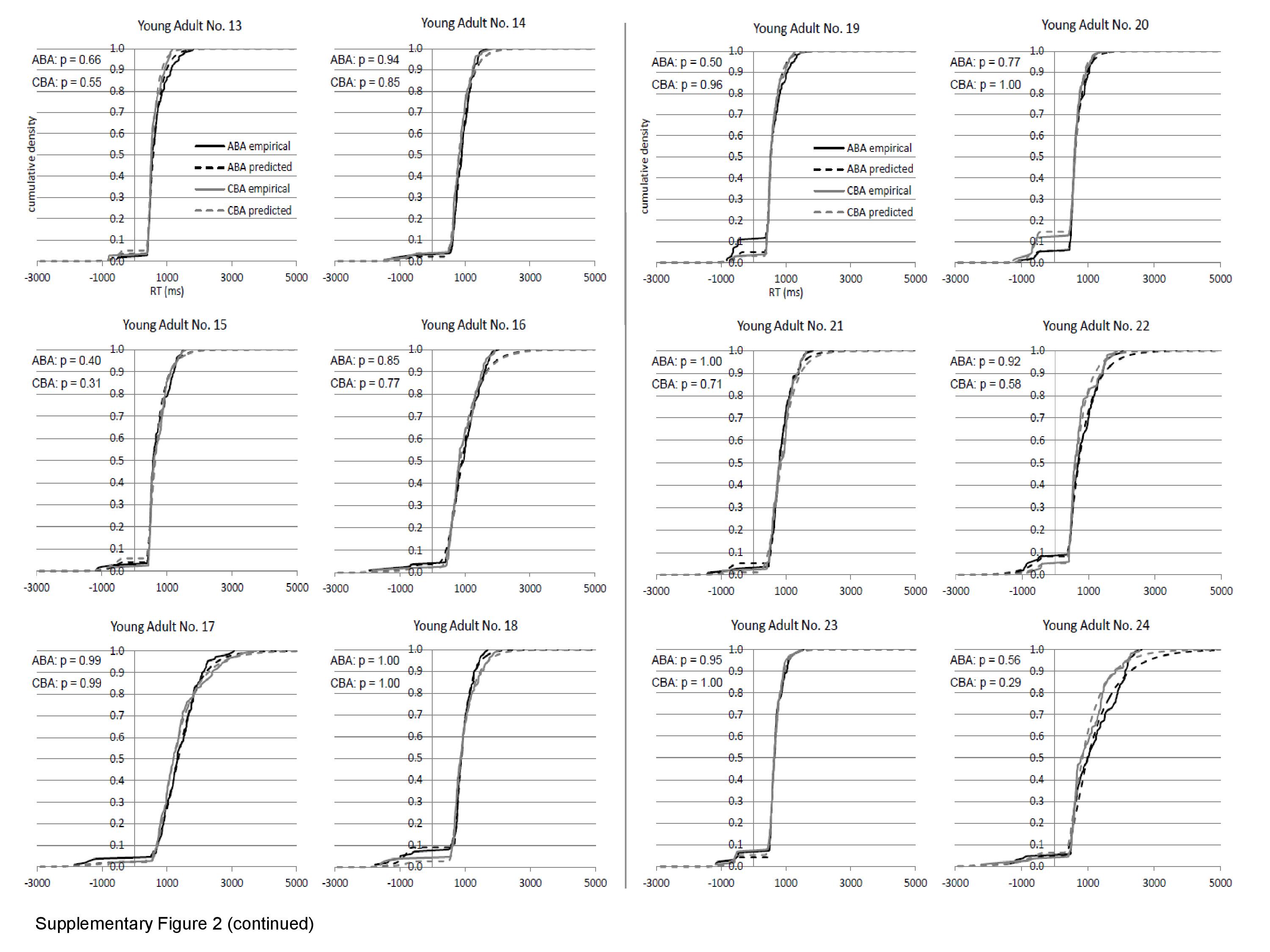

Supplement: Supplementary file 6 [file Image6.JPEG]

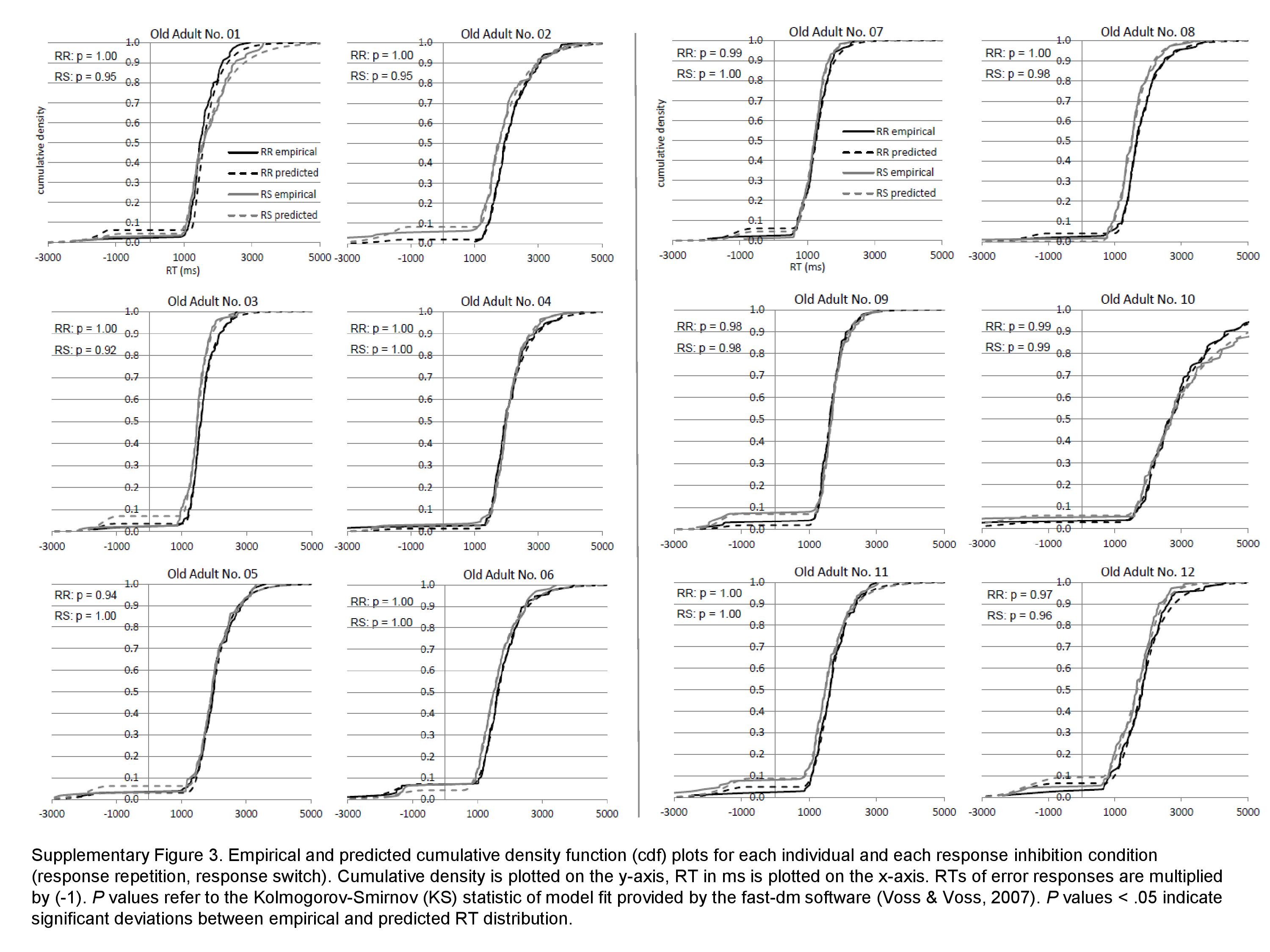

Supplement: Supplementary file 7 [file Image7.JPEG]

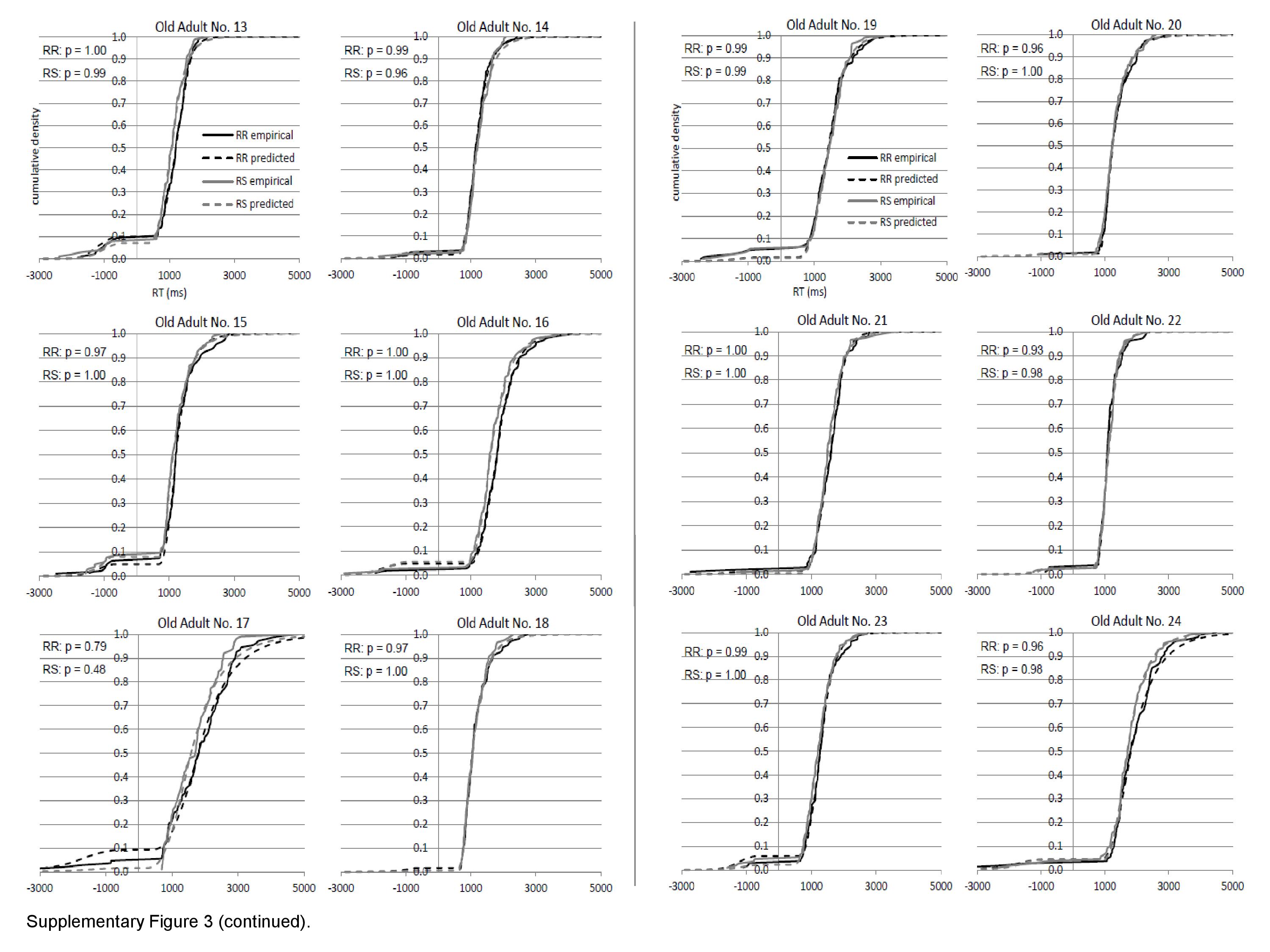

Supplement: Supplementary file 8 [file Image8.JPEG]

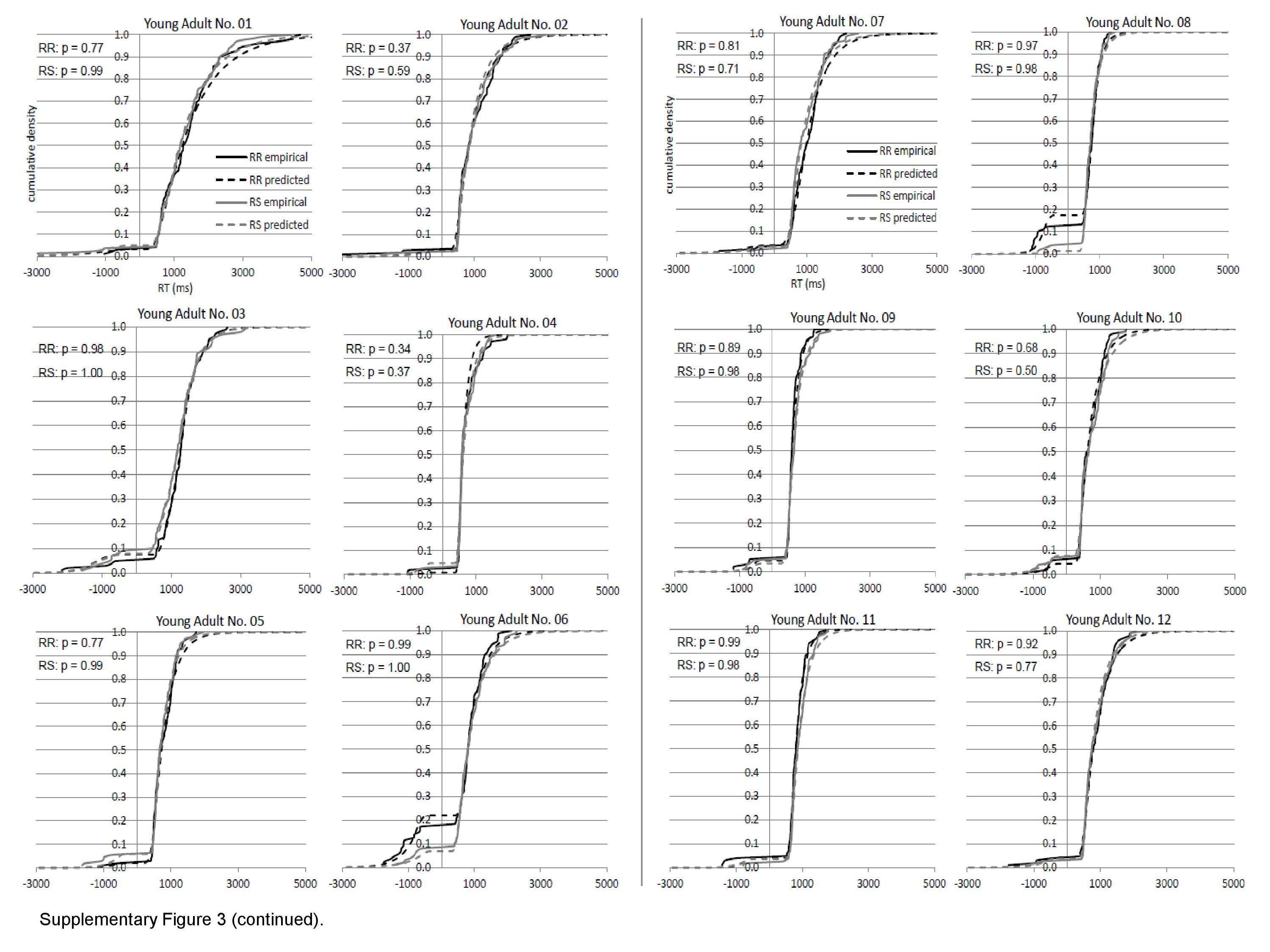

Supplement: Supplementary file 9 [file Image9.JPEG]

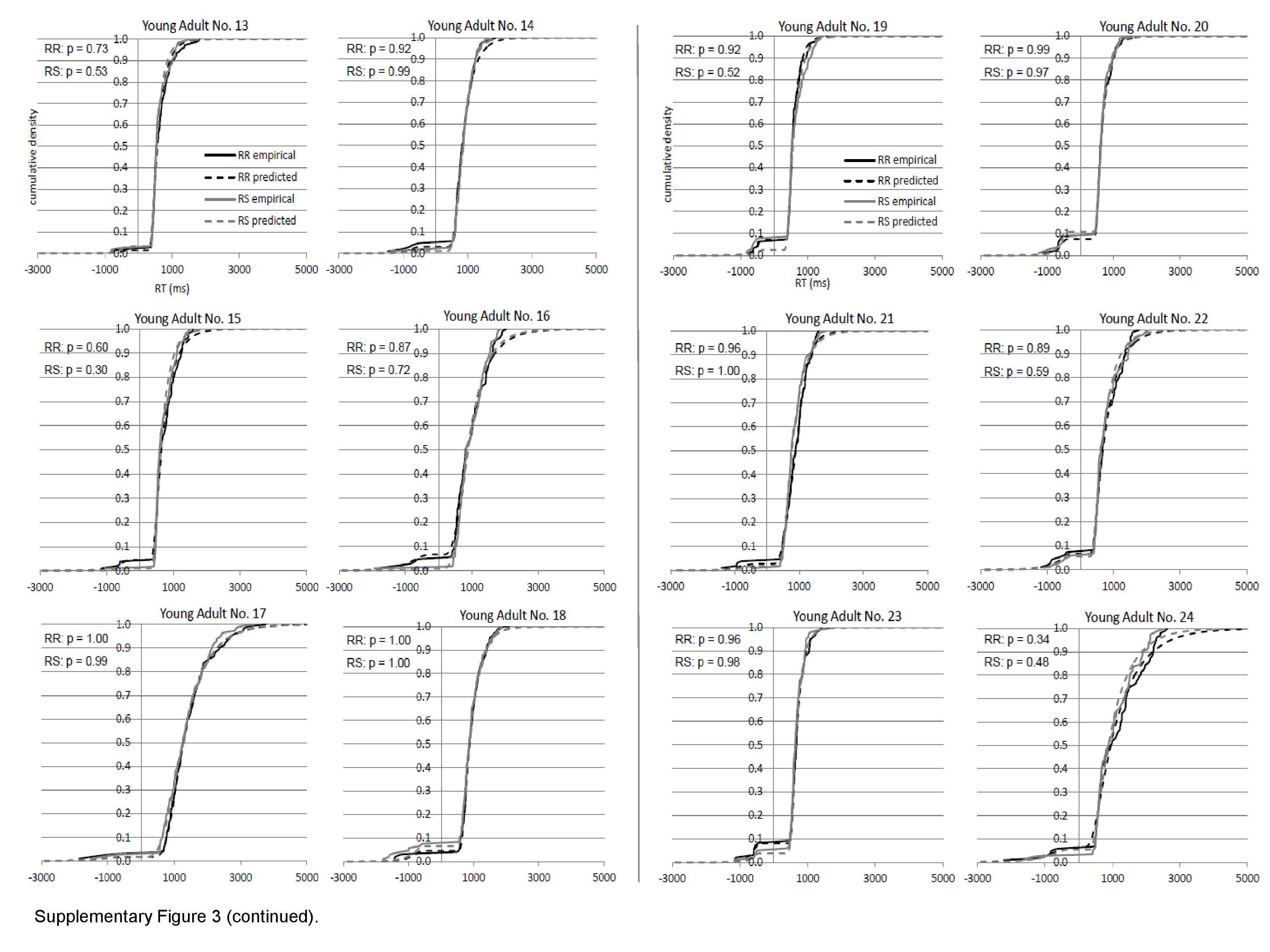

Supplement: Supplementary file 10 [file Image10.JPEG]
